# Validation of a Curricular Map on Cardiac Implantable Electronic Devices (CIEDs) for Cardiac Anesthesia Trainees

**DOI:** 10.7759/cureus.98104

**Published:** 2025-11-29

**Authors:** Ahmed F. Zaky, Brittany Hatter, Srilakshmi Malempati, Sai Hemanth Maremalla, Ragib Hasan, Scott Snyder

**Affiliations:** 1 Anesthesiology, University of Alabama at Birmingham, Birmingham, USA; 2 Anesthesiology and Perioperative Medicine, University of Alabama at Birmingham, Birmingham, USA; 3 Computer Science, University of Alabama at Birmingham, Birmingham, USA; 4 Education, University of Alabama at Birmingham, Birmingham, USA

**Keywords:** anesthesia trainees, cardiac anesthesia, cardiac implantable electronic device (cied), curriculum mapping, validation

## Abstract

Introduction: Despite an increased exposure of cardiac anesthesia trainees to cardiac implantable electronic devices (CIEDs), there is a paucity of formal curricula on the subject. Herein, we present the validation of a CIED curricular map by a group of CIED experts and educators. The purpose of this stage of curriculum development is to determine whether experts in education regarding CIEDs consider: (a) the goals and objectives to be critical, (b) the degree to which the instructional materials align with specific objectives, (c) the degree to which the assessments reflect the objectives to which they have been aligned, and (d) the degree to which the assessments are adequately informed by the instructional materials for the aligned objectives.

Methods: The curriculum validation process involved the identification of a national panel of 15 experts in curricula relating to CIEDs. Each panelist received an online survey (Qualtrics), which presented the 17 major learning goals of the curriculum and the instructional objectives for each goal (5-15 objectives per goal). Respondents were asked to rate the importance of each instructional objective (not important, important, and essential), the fit of the objective to the learning goal (does not fit and aligns), and the clarity of the objective (unclear/ambiguous and clear). Respondents were also asked to propose additional objectives that would be needed to adequately represent the learning goal. A content validity ratio (CVR) is computed for each instructional objective based on the proportion of panelists who rate the objective as essential. A content validity index (CVI) is computed for each learning goal based on the sum of the CVRs for the objectives comprising a goal divided by the number of panelists. CVIs of greater than 0.72 across at least three raters are considered to provide sufficient evidence of the validity. Other variables (fit, clarity, and supplemental objectives) within the survey were used to discern and improve the quality of the objectives.

Results: While all objectives were rated as important or essential by at least 80% of the 15 raters, several goals had fewer than 70% of their objectives rated as essential by 73% or more of the experts. Notably, six goals (3, 6, 8, 10, 12, and 13) had no objectives rated as essential by more than 73% of the experts. Although the overall CVR and CVI were lower than desired, there was significant agreement regarding the importance of the objectives for each goal. More than 80% of experts indicated that all objectives appropriately fit their respective goals.

Conclusions: CIED curriculum is well-received by national experts. Curricular refinements were made to meet the desired goals of agreement before finalizing the curriculum.

## Introduction

The number of patients with cardiac implantable electronic devices (CIEDs) is on the rise, with approximately 400000 devices implanted annually [1]. Due to increased longevity and improvement in medical care, a great proportion of this population is undergoing surgical procedures [2]. This has created a need for anesthesiologists to gain the skills and knowledge to manage these devices [3].

Cardiac anesthesiology (CA) trainees include fellows who undergo a year-long training in an Accreditation Council for Graduate Medical Education (ACGME)-accredited cardiothoracic anesthesiology fellowship program, as well as residents at different stages of their four-year categorical training who rotate for one or two months in the division of cardiothoracic anesthesiology. Mastery in managing patients with CIEDs requires a strong foundation in electrophysiology, general cardiac physiology, types and operation of CIEDs, and their interactions with electrosurgical magnetic interferences and with other cardiac devices such as ventricular assist devices and extracorporeal membrane oxygenators.

Despite increased exposure of CA trainees to CIEDs, the existence of published guidelines, and calls for a CIED curriculum, no formal curricula exist to educate them on this subject [4-6]. Although literature is available regarding the benefits of various types of CIED-related training, no published research was found that outlines specific educational goals and learning objectives for a curriculum [7]. Furthermore, current educational materials on the subject lack formal validation by a group of content experts, do not quantify learning gains or experiences, and are collectively insufficient to build and sustain competency in managing rapidly evolving CIEDs in sick patients [3,8].

To bridge this gap, we created a comprehensive curriculum that targets CA trainees and ought to validate the alignment of its goals and objectives through a nationwide survey of subject matter experts (SME) in the field.

## Materials and methods

The study was approved by the University of Alabama Institutional Review Board and adheres to the applicable CONSORT Guidelines. The requirement for patient consent was waived.

Curriculum design and development

Curriculum development involved the identification of broad goals representing core content areas within textbooks, supplemented by additional areas identified by experts in the CIED field. The lead author (AZ) generated learning objectives that reflected the breadth and scope of each goal. The lead author is a trained cardiac anesthesiologist and intensivist with special expertise in the perioperative management of CIEDs. He is also the director of an anesthesiology-led clinical service at the institution [9]. A critical component of curriculum validation involves evaluating the importance and quality of the objectives that provide the structure for the curriculum, as well as subsequent instruction and assessment. High-quality objectives are those that SEMs consider: (a) critical in representing the goal, (b) logically aligned with the goal, and (c) clearly written to improve their utility in guiding instruction and assessment.

**Figure 1:**
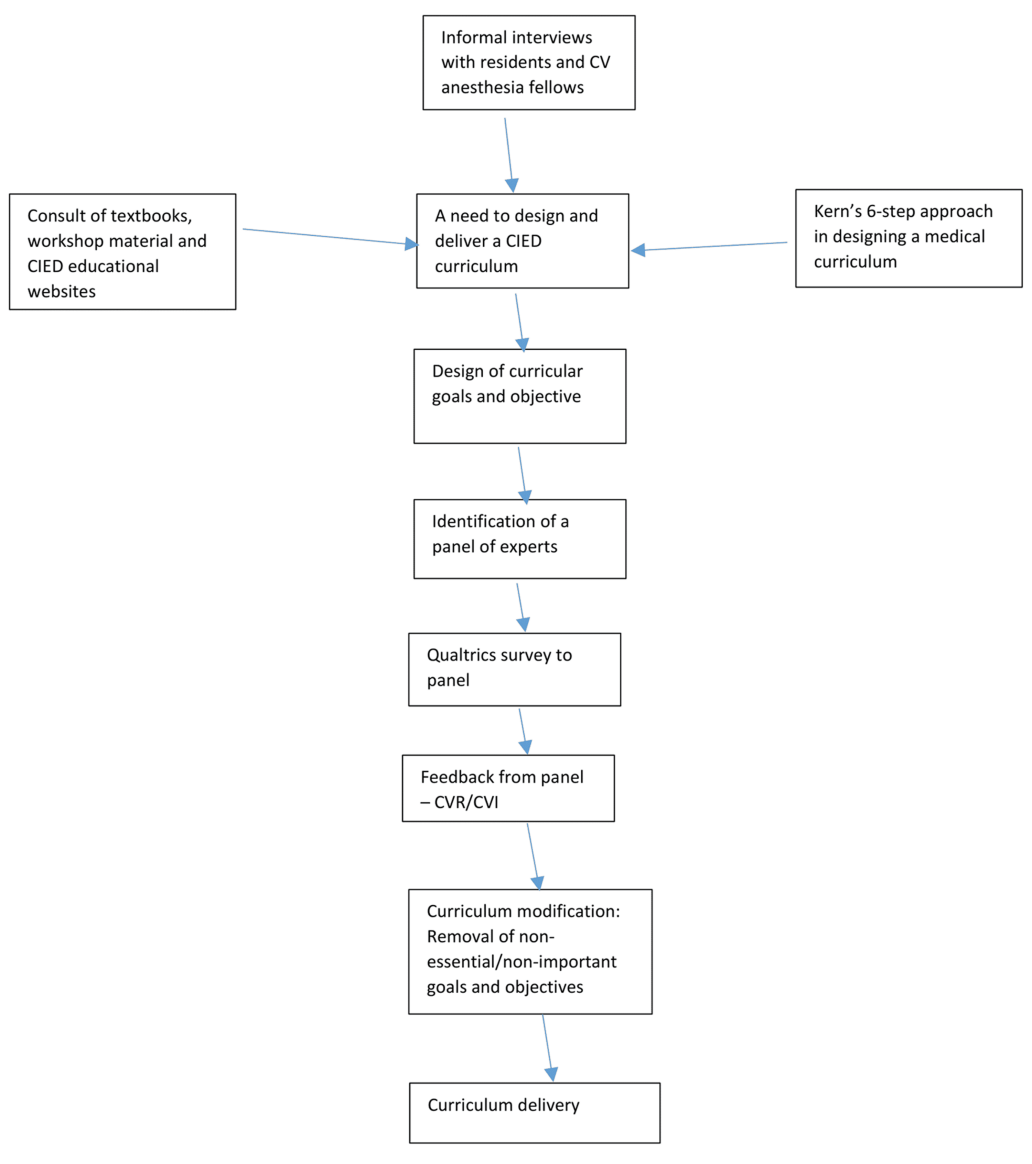
Flow chart of curricular design and delivery process CIED: cardiac implantable electronic device; CVR: content validity ratio; CVI: content validity index; CV: cardiovascular Image credit: authors

Following the development of an initial pool of 15 goals and their associated objectives, we sought to evaluate the proposed goals and objectives by surveying a group of CIED experts regarding whether (a) the objectives are critical or important to CA learners, (b) the instructional objectives align with the learning goals, and (c) the objectives, as written, are clear. After the initial development of the goals and objectives, the process for evaluating the objectives was similar to methods employed in a previous medical curriculum validation process used in Obstetrics in Canada [10]. A flow chart of the curricular design and validation process is outlined in Figure 1.

Curriculum validation

The curriculum validation process involved the identification of a national panel of experts in curricula related to CIEDs. The definition of an expert was based on the following qualifications: board certification in Cardiology, Electrophysiology, or an equivalent CIED-related certification; more than five years of experience; and expertise in medical curriculum design.

Each panelist received an online survey (Qualtrics) that presented the 15 major learning goals of the curriculum and the instructional objectives for each goal (2-15 objectives per goal). Respondents were asked to rate the importance of each instructional objective (not important, important, and essential), the fit of the objective to the learning goal (does not fit and aligns), and the clarity of the objective (unclear/ambiguous and clear). Two content validity ratios (CVR) were computed for each instructional objective. CVRs were calculated according to the formula "/(CVR: (ne-N/2)÷N/2"/), where ne: number of experts who determined that the goal is "essential" and N: total number of experts. For the purposes of this analysis, the ratios reflected either the proportion of panelists rating an objective as "essential" or the percentage of panelists rating the objective as "important" or "essential". Essential objectives were considered absolute requirements to include in a curriculum. Important objectives reflected those outcome expectations that the experts believed were valuable but not essential to include in the curriculum. The CVR for "essential" ratings was termed stringent, whereas the CVR for "essential or important" ratings was termed modest. Traditionally, CVRs have been used to determine whether individual items match instructional objectives [11]. Items whose CVR for those considered essential to measuring the objective was below a criterion level based on the number of raters were excluded from inclusion. As this analysis does not relate to individual items but rather to a comprehensive curriculum related to CIEDs, the authors evaluated the CVRs in regard to the degree to which objectives were considered: (a) essential based on the goal and (b) either essential or important. Based on the number of raters, the criterion recommended for inclusion of items was a CVR of 0.75 [11,12].

In addition, content validity indices (CVIs) were computed for each learning goal based on the average of the CVRs for the objectives. Based on the criteria proposed by Ayre et al. [12], a CVI greater than 0.78 was considered to provide sufficient evidence of the validity of the goal [13]. Experts were also asked to judge whether the objectives were a good fit for the goal and whether the objectives were clearly stated.

## Results

A total of 17 out of 21 SMEs responded to the survey: five electrophysiologists, four CIED vendor representatives, seven general cardiologists, and one anesthesiologist. Most of the experts worked at academic centers, with only one expert working at a private institution. Four of the 17 panelists were from the authors’ institution.

Twenty-one of the 77 objectives were considered “essential,” and hence stringent by CVI, by 75% or more of the raters. Those objectives are clustered into eight goals. Seven of the 15 goals had no objectives that met the criterion of being “essential” to 75% or more of raters (goals 3, 6, 8, 10, 12, 13, and 15). The only goal for which the average rating of objectives as “essential” or “important,” and hence modest by CVI, was less than 0.9 across raters was “understand the pertinent unique features of each of the four CIED vendors” (Appendix provides the results of the ratings of the 17 judges regarding the importance, fit, and clarity of the objectives).

There were four goal areas that met the most stringent criteria of having average CVI indices of over 0.75 based on ratings that the objectives were “essential,” and having average CVI indices at or above 0.9 based on ratings that the objectives were rated as “important” or “essential” (Table 1). All other goals, with the exception of “understanding the unique features of each of the four CIED vendors,” demonstrated average CVI ratings below 0.75 for objectives rated as essential, but had average ratings of 0.9 or higher for objectives rated as “important” or “essential.”

**Table 1:** Goals with high CVR objectives by SMEs Based on the number of raters, the criterion recommended for inclusion of items was a CVR of 0.75. CVIs were computed for each learning goal based on the average of the CVRs for the objectives. A CVI greater than 0.78 was considered to provide sufficient evidence of the validity of the goal. CVR: content validity ratio; CRT: cardiac resynchronization therapy; SQ: subcutaneous; ICD: implantable cardioverter; CIEDs: cardiac implantable electronic devices; CVI: content validity index; SME: subject matter expert

	CVR "modest" <0.9	CVR “modest" ≥0.9
CVR “stringent” <0.75	Understand unique features of each of the four CIED vendors (0.89)	Understand the indications for placement of different CIEDs, understand the hemodynamic effects of cardiac pacing, understand the modes of CIED pacing, understand upper rate behavior of pacemakers, understand the different components of a permanent pacemaker, understand the indications, function, and operation of a temporary pacemaker, understand the indications, operation, and structure of leadless pacemakers, understand the basic structure and function of ICDs, understand the clinical utility of the SQ ICD, and demonstrate a basic understanding of the clinical operations of CRTs
CVR “stringent” at ≥0.75		Introductory guide to cardiac electrophysiology, interpret and recognize basic pacemaker timing cycles, understand pacer malfunction and pseudo-malfunction, and understand common features shared by all four vendors of CIEDs

Seven goals had no objectives that met the "stringent" criterion of being rated as “essential” by 75% or more of the SMEs. However, four of these goals had all objectives rated as “modest” by 90% or more of SMEs, and two goals had only one objective that was not rated as “modest” by 90% or more of SMEs.

The objectives that were considered to be “modest” by fewer than 90% of raters were: 1. Identify the action potentials of different components of the cardiac conduction system (0.88); 2. Compare and contrast the VVI mode of leadless versus conventional pacemakers (0.87); 3. Discriminate the rate modulation terminology in Biotronik (0.75); 4. Discriminate the upper rate behavior of Biotronik (0.82); 5. Discriminate the upper rate behavior of Medtronic (0.82); 6. Discriminate the automatic mode switching algorithm of Medtronic (0.88); 7. Discriminate the upper rate behavior and mode switching algorithm of SJM (0.81); and 8. Discriminate the mode switching algorithm of BS (0.82) (Table 2).

**Table 2:** Examples of goals not meeting stringent or modest criteria by 90% of raters ICD: implantable cardioverter defibrillators; BS: Boston Scientific; SJM: St. Jude Medical; VDD: ventricular paced, dual chamber sensed, pacing inhibition by respective chamber's activity, intrinsic activity triggers pacing of corresponding chamber

Objective	% not important	% important	% essential
Discriminate rate modulation terminology in Biotronik	25	44	31
Discriminate the VDD pacer configuration of Biotronik	6	62	31
Discriminate upper rate behavior of Biotronik	19	44	38
Discriminate the magnet rate of Biotronik	6	38	56
Discriminate magnet interaction with Medtronic ICDs and pacers	6	38	56
Discriminate the upper rate behavior of Medtronic pacers	19	44	38
Discriminate the automatic recode switching algorithm of Medtronic	12	50	38
Discriminate the magnet rate of the SJM pacer	6	44	50
Discriminate the upper rate behavior and mode switching algorithm of SJM	19	50	31
Discriminate the magnet rate of BS pacer	6	44	50
Execute activation of the electrocautery function	0	31	69
Discriminate mode switching algorithm of D5	19	38	44

Ratings of fit and clarity of all objectives were high. The only objective that was rated as not fitting the goal by 85% or more of the raters was “execute atrioventricular synchrony in patients with hypertrophic cardiomyopathy” within the “understand the hemodynamic effects of cardiac pacing” goal. That objective was viewed as a fit by 75% of experts. Four objectives were considered clear by fewer than 80% of the experts. Those objectives were: 1. Execute atrioventricular synchrony in patients with hypertrophic cardiomyopathy (75%), 2. List pacemaker codes (75%), 3. Identify the difference between CRT-S and CRT-P (53%), and 4. Recognize battery failure EKG patterns (73%).

## Discussion

To our knowledge, this is the first validation study of a curriculum on the subject of CIEDs for a specialty trainee. The results of this study provide tentative validation of the proposed curriculum for CIEDs, as well as insight into the strengths and weaknesses of the proposed curriculum.

The results highlight two key findings. First, a significant proportion of objectives met established criteria for being “important or essential” but not “essential.” Second, most goals met the validation criterion. For example, the objective “Recognize how to optimize atrioventricular synchrony” was considered to align with the goal “Understand the hemodynamic effects of cardiac pacing” and was clear to all raters, but was rated as essential by only 41% of the SMEs. However, it was rated as “important” by the other 59% of SMEs. Therefore, while fewer than half of the SMEs considered the objective “essential,” 100% considered the objective to fit, be clear, and be either important or essential in reflecting the goal. Only “Understand the pertinent unique features of each of the four CIED vendors” had more than one objective (50%) that was not rated as “important or essential” by more than 90% of the SMEs. These objectives tended to focus on upper-rate behavior and/or mode-switching algorithms of the various CIED vendors. SMEs justified their underrating of these objectives due to, first, the availability of device representatives who are most familiar with these algorithms and can be consulted both at implantation and in clinic settings; second, the constantly changing nature of these algorithms, which may outpace the learning capacity of trainees; and third, the relatively out-of-scope nature of these algorithms for the cardiac anesthesiologist.

The objective of “maintaining AV synchrony in patients with hypertrophic obstructive cardiomyopathy” was rated low by a large proportion of SMEs due to the controversy surrounding this subject. Atrioventricular sequential pacing with a short AV delay was thought in the past to reduce left ventricular outflow obstruction and reduce symptoms in patients with HOCM [14]. However, recent evidence from large meta-analyses [15] and systematic reviews has questioned the superiority of this pacing modality in comparison to septal myectomy and alcohol septal ablation, the primary invasive treatment modalities [16,17]. The SMEs felt that a controversial subject may not be suitable for CA trainees. Another goal that was considered clear by less than 80% of SMEs was “EKG patterns of CIED battery depletions”. SMEs felt that the goal may not be important since CIED battery depletion could be detected prior to EKG changes, and there are no pathognomonic EKG changes that are diagnostic of CIED battery depletion. For the other two goals, list pacemaker codes and recognize the difference between CRT pacing (CRT-P) and CRT with defibrillator capability (CRT-D), SMEs expressed that the learning objective wording needed to be changed and that discussing the differences between CRT-P and CRT-D is repetitive because the difference between pacing and defibrillators in general was discussed in a prior module. One objective, “comparing pacemaker modes between leadless and transvenous pacemakers”, was considered too advanced for CA trainees by SMEs.

The seven goals in the survey that had no objectives meeting the criterion of being rated as "essential" by 75% of the SMEs could reflect either a concern among the reviewers regarding the importance of the goal itself or that there were no specific objectives considered "essential" for representing the goal. The latter explanation is supported by evidence that 75% or more of the 17 judges rated all objectives in the proposed curriculum as either "important" or "essential" in fitting the goal, including the objectives within the seven goals that did not meet the criterion for "essential."

Our choice of SMEs is one of the strengths of the study. As such, it is in accordance with what Kern and colleagues have proposed to provide validity for a curriculum [18]. We selected a diverse group of SMEs that span the fields of general cardiology, electrophysiology, anesthesiology, and device education, which provides robustness to the validation process. The use of interdisciplinary SMEs has been employed in similar curricula in medical education [19-21]. The inclusion of non-anesthesiologist SMEs in an anesthesiology-related curriculum, while it may be viewed as a limitation, is also a strength. Non-anesthesiologist SMEs expand the scope of the curriculum and, hence, the learning benefit gained by anesthesiology trainees. Additionally, non-anesthesiologist SMEs, such as device representatives and cardiologists, are constantly engaged in the perioperative management of CIEDs and are familiar with anesthesiology-appropriate curricular content. Furthermore, we chose SME experts who work in different hospital settings, an advantage that will serve trainees who are expected to work in the community. The inclusion of four internal SMEs poses a potential bias; however, we believe that the inclusion of internal SMEs is crucial for internal validation of the curriculum. The inclusion of internal and external SMEs thus provides a form of simultaneous internal and external validation of the curriculum. Additionally, our internal SMEs were the most critical of the curricular goals and objectives. 

Our study has some limitations. While this study included a sufficient number of SMEs, the lack of precision in operationalizing differences between objectives considered “essential” and those considered “important” may have compromised the precision and reliability of the CVIs and CVRs. Another limitation is that there was no exploration of goals that other SMEs may have considered critical to include in the proposed curriculum. At the time of writing this manuscript, the validation process encompassed only the alignment between the goals and objectives. The authors have modified the curricular goals and objectives by removing those that did not conform to the criteria of clarity or importance. Curricular content that was considered out of scope or too advanced for CA trainees was compiled to create an advanced curriculum for cardiology trainees. A pilot trial of the curriculum was delivered to trainees through a dedicated application. We are currently collecting data on learning gains and the usability and likeability of the app by our trainees [22]. Data collected from the pilot trial will be added to data obtained from a new validation survey that assesses the alignment of the modified curricular objectives with the instructional and assessment material. Together, these data will serve as the basis for the next phase of the curriculum.

## Conclusions

The curriculum on CIEDs was well received by SMEs. Important modifications were made to improve the quality of the curriculum. Subsequent research is recommended to examine the adequacy of the breadth of the goals, the qualitative reasons for differentiation between “essential” and “important” objectives within this and other medical specialty domains, and the role of medical curricula in preparing practitioners to respond to differences in the unique features of CIEDs and other medical devices. Delphi surveys and interviews are likely strategies to achieve these goals.

## Appendices

**Table 3:** Evaluation of importance, fit, and clarity of proposed CIED curriculum objectives ^*^The percentage of experts who deem the objective “essential” over the percentage of experts who deem the objective “important.” ^#^Numerator is the average of the percentage of raters who rated this goal as essential. The denominator is the average of the percentage of raters who rated this goal as essential or important. The higher each of these numbers is, the higher the perceived average of this objective within the goal. CVR: content validity ratio; CRT: cardiac resynchronization therapy; SQ: subcutaneous; ICD: implantable cardioverters; CIEDs: cardiac implantable electronic devices; SJM: St. Jude Medical; VDD: ventricular paced, dual chamber sensed, pacing inhibition by respective chamber's activity, intrinsic activity triggers pacing of corresponding chamber; BS: Boston Scientific; CRT-D: cardiac resynchronization therapy-defibrillator; CRT-P: cardiac resynchronization therapy-pacing; DDD: dual chamber paced, dual chamber sensed, pacing-inhibited by respective chamber activity, tracking of intrinsic activity of corresponding chamber; PMT: pacemaker-mediated tachycardia; VVI: ventricular paced ventricular sensed and pacing inhibition by intrinsic ventricular activity

Goal	Objective	Importance of objective (% essential/% important)	Fit of objective (% agreeing that the objective aligns)	Clarity of objective (% rating the objective as clear)
To provide an introductory guide to cardiac electrophysiology (CVI=0.80/0.96^#^)				
	Identify the action potentials of different components of the cardiac conduction system	41/47^*^	100	88
	Recognize surface EKG patterns of different types of heart blocks	100	100	100
	Recognize surface EKG patterns of supraventricular and ventricular tachycardias	100	100	100
To apply knowledge and skills learned from module 1 to understand the indications of placement of different CIEDs (CVI-0.72/1.00)				
	Classify the different indications of permanent pacemaker placement	71/29	100	94
	List the indications of placement of ICDs	71/29	100	94
	List the indications of placement of CRTs	65/35	100	94
	Recognize surface EKG patterns pertinent to the indications of individual CIED placement	81/19	94	88
To apply knowledge and skills acquired from previous modules to understand the hemodynamic effects of cardiac pacing (CVI=0.63/0.97)				
	Recognize how to optimize atrioventricular synchrony	41/59	100	100
	Recognize the drawbacks of right ventricular pacing	53/41	94	94
	Identify strategies to avoid right ventricular pacing	59/35	94	94
	Recognize how rate modulation works	53/41	94	94
	Recognize the cause, manifestations, and treatment of pacemaker syndrome	47/53	100	100
	Execute atrioventricular synchrony in patients with hypertrophic cardiomyopathy	41/53	75	75
	Execute atrioventricular synchrony in complete heart block	56/44	87	87
To apply knowledge and skills learned in previous modules to understand the modes of CIED pacing (CVI=0.57/0.96)				
	List pacemaker codes	47/47	87	75
	List pacemaker modes	76/24	100	100
	List ICD codes	50/44	93	75
	List CRT codes	53/41	93	81
Applying what is learned in the basics section to interpret and recognize basic pacemaker timing cycles (CVI=0.76/1.00)				
	Recognize pacemaker nomenclature	88/12	100	87
	Recognize different pacemaker modes	94/6	100	93
	Identify different portions of the pacemaker cycle	63/37	100	86
	Identify timing cycles for biventricular pacer	63/37	100	100
	Identify the 4 faces of DDD pacing	73/27	100	87
Understanding upper rate behavior of pacemakers (CVI=0.51/0.96)				
	Identify the different patterns of upper rate behavior	47/47	93	100
	Identify mode switching algorithms	53/41	93	100
	Identify sensor-based algorithms	47/47	87	94
	Identify the electrical basis of endless loop tachycardia	59/41	100	88
	Differentiate atrial-based versus ventricular-based behaviors	47/53	100	94
Understand pacer malfunction and pseudo-malfunction (CVI=0.78/0.97)				
	Recognize failure to capture	100	100	100
	Recognize failure to sense	100	100	100
	Recognize failure to pace	100	100	100
	Recognize battery failure EKG patterns	69/31	93	73
	Interpret noise pseudo-malfunction	81/19	100	80
	Interpret electrocautery-related malfunction	81/19	100	86
	Differentiate between different causes of oversensing	75/25	100	100
	Differentiate between different causes of open circuit	67/27	93	87
	Identify hysteresis, PMT, and mode switching as causes of pseudo-malfunction	69/31	100	87
To understand the different components of a permanent pacemaker (CVI=0.49/0.95)				
	Recognize the different types of a permanent pacemaker leads	25/69	87	87
	Recognize the make and function of pulse generators	50/44	93	87
	Differentiate between unipolar and bipolar pacing	69/31	100	100
	Recognize reed switch and magnet function	69/31	100	93
	Recognize radiological identification of pacemakers	31/56	87	87
To understand the indications, function, and operation of temporary pacemakers (CVI=0.73/0.98)				
	Identify the different routes of temporary pacemaker placement	63/31	100	93
	List the indications of temporary pacemaker placement	75/25	100	100
	Identify and troubleshoot temporary pacemaker malfunction	81/19	100	100
To understand the indications, operation, and structure of leadless pacemakers (CVI=0.49/0.97)				
	Illustrate the indications of leadless pacemakers	27/67	93	86
	Discriminate magnet effects on conventional versus leadless pacemakers	67/33	100	93
	Compare and contrast the VVI mode of leadless versus conventional pacemakers	40/47^*^	100	86
	Compare and contrast complications of leadless versus conventional pacemaker	60/40	100	100
To understand the basic structure and function of ICDs (CVI=0.63/1.00)				
	Describe how the ICD detects, discriminates, and treats ventricular arrhythmias	81/19	100	93
	Discriminate radiological appearance of ICDs from pacemakers	44/56	87	93
Understand the clinical utility of SQ ICD (CVI=0.54/0.98)				
	Identify the indications of SQ ICD	50	92	92
	Compare and contrast the operation of SQ ICD vs. conventional CID.	50	100	100
	Describe the complications associated with SQ ICD	57	100	100
Gain a basic understanding of the clinical operations of CRTs (CVI = 0.48/0.95)				
	Identify the differences between CRT-S and CRT-P	29/64	85	53
	Understand the individual components of the CRTs	54/40	92	80
	Describe the optimal position of placing the LV lead	50/44	93	100
	Differentiate univentricular versus biventricular pacing	56/38	93	80
	Describe the challenges with placement of CRT leads	50/50	100	100
Understand common features of all 4 vendors of CIEDs (CVI=0.76/0.99)				
	Describe the 4 screens pertinent to all programmers	75/25	93	80
	Execute better interrogation testing on all programmers	81/13	100	93
	Execute pacing threshold testing on all programmers	81/19	100	100
	Execute sensing amplitude testing on all programmers	81/19	100	100
	Execute lead impedance testing on all programmers	81/19	100	100
	Define pacer dependency in	81/19	100	93
Understand the pertinent unique features of each of the 4 CIED vendors (CVI=0.41/0.89)				
	Discriminate the rate modulation terminology in Biotronik	31/44^*^	93	100
	Discriminate the VDD pacer configuration of Biotronic	31/63	87	86
	Discriminate the upper rate behavior of Biotronic	38/44^*^	87	100
	Discriminate magnet rate of Biotronic	56/38	93	100
	Discriminate magnet interaction with Medtronic ICDs and pacers	56/38	93	93
	Discriminate the upper rate behavior of Medtronic	38/44^*^	87	93
	Discriminate the automatic mode switching algorithm of Medtronic	38/50^*^	93	93
	Discriminate magnet rate of SJM pacer	50/44	87	93
	Discriminate the upper rate behavior and mode switching algorithm of SJM	31/50^*^	87	93
	Discriminate the magnet rate of BS pacer	50/44	87	93
	Execute activation of the electrocautery function	69/31	80	93
	Discriminate mode switching algorithm of BS	44/38^*^	77	92

## References

[1] Kremers MS, Hammill SC, Berul CI (2013). The National ICD registry report: version 2.1 including leads and pediatrics for years 2010 and 2011. Heart Rhythm.

[2] (2020). Cardiac implantable electronic device management [corrected]. Anesthesiology.

[3] Thorpe RL, Rohant N, Cryer M, Gainey C (2019). Inappropriately firing defibrillator: a simulation case for emergency medicine residents. MedEdPORTAL.

[4] American Society of Anesthesiologists (2011). Practice advisory for the perioperative management of patients with cardiac implantable electronic devices: pacemakers and implantable cardioverter-defibrillators: an updated report by the American Society of Anesthesiologists task force on perioperative management of patients with cardiac implantable electronic devices. Anesthesiology.

[5] Crossley GH, Poole JE, Rozner MA (2011). The Heart Rhythm Society (HRS)/American Society of Anesthesiologists (ASA) Expert Consensus Statement on the perioperative management of patients with implantable defibrillators, pacemakers and arrhythmia monitors: facilities and patient management: executive summary this document was developed as a joint project with the American Society of Anesthesiologists (ASA), and in collaboration with the American Heart Association (AHA), and the Society of Thoracic Surgeons (STS). Heart Rhythm.

[6] Zipes DP, Calkins H, Daubert JP (2016). 2015 ACC/AHA/hrs advanced training statement on clinical cardiac electrophysiology (a revision of the ACC/AHA 2006 update of the clinical competence statement on invasive electrophysiology studies, catheter ablation, and cardioversion). Heart Rhythm.

[7] Özkartal T, Demarchi A, Caputo ML, Baldi E, Conte G, Auricchio A (2022). Perioperative management of patients with cardiac implantable electronic devices and utility of magnet application. J Clin Med.

[8] Smith AW, Elliott JO, Gable BD (2021). Simulation improves internal medicine resident confidence with defibrillation, cardioversion, and transcutaneous pacemaker use. Cureus.

[9] Zaky A, Beck A, Rooke GA (2020). Hemodynamically significant heart block after carotid artery stenting in a patient with atrial demand pacer-echocardiography-guided rescue pacing. J Cardiothorac Vasc Anesth.

[10] Cumyn A, Harris IB (2012). A comprehensive process of content validation of curriculum consensus guidelines for a medical specialty. Med Teach.

[11] Lawshe CH (1975). A quantitative approach to content validity. Pers Psychol.

[12] Ayre C, Scally AJ (2014). Critical values for Lawshe’s content validity ratio: revisiting the original methods of calculation. Meas Eval Couns Dev.

[13] Almohanna AA, Win KT, Meedya S, Vlahu-Gjorgievska E (2022). Design and content validation of an instrument measuring user perception of the persuasive design principles in a breastfeeding mHealth app: a modified Delphi study. Int J Med Inform.

[14] Fananapazir L, Epstein ND, Curiel RV, Panza JA, Tripodi D, McAreavey D (1994). Long-term results of dual-chamber (DDD) pacing in obstructive hypertrophic cardiomyopathy. Evidence for progressive symptomatic and hemodynamic improvement and reduction of left ventricular hypertrophy. Circulation.

[15] Arnold AD, Howard JP, Chiew K (2019). Right ventricular pacing for hypertrophic obstructive cardiomyopathy: meta-analysis and meta-regression of clinical trials. Eur Heart J Qual Care Clin Outcomes.

[16] Qintar M, Morad A, Alhawasli H, Shorbaji K, Firwana B, Essali A, Kadro W (2010). Pacing for drug-refractory or drug-intolerant hypertrophic cardiomyopathy. Cochrane Database Syst Rev.

[17] Elliott PM, Anastasakis A, Borger MA (2014). 2014 ESC guidelines on diagnosis and management of hypertrophic cardiomyopathy: the task force for the diagnosis and management of hypertrophic cardiomyopathy of the European Society of Cardiology (ESC). Eur Heart J.

[18] Kern DE, Thomas PA, Hughes MT, Chen BY, Tackett SA (2022). Curriculum Development for Medical Education: A Six-Step Approach. Baltimore, Md.: Johns Hopkins University Press.

[19] Castleberry AN, Schneider EF, Carle MH, Stowe CD (2016). Development of a summative examination with subject matter expert validation. Am J Pharm Educ.

[20] Ambardekar AP, Eriksen W, Ferschl MB, McNaull PP, Cohen IT, Greeley WJ, Lockman JL (2023). A consensus-driven approach to redesigning graduate medical education: the pediatric anesthesiology Delphi study. Anesth Analg.

[21] DeLellis T, Noureldin M, Park SK, Shields KM, Bryant A, Chen AM, Petrelli HM (2022). A situational judgment test to assess students’ achievement of Accreditation Council for Pharmacy Education standards 3 and 4. Am J Pharm Educ.

[22] Zaky A, Waheed A, Hatter B (2025). Cardiac implantable electronic device educational application for cardiac anesthesiology trainees: tutorial on app development. JMIR Med Educ.

